# Diagnostic Role of **^18^**F-FECH-PET/CT Compared with Bone Scan in Evaluating the Prostate Cancer Patients Referring with Biochemical Recurrence

**DOI:** 10.5402/2012/815234

**Published:** 2012-11-28

**Authors:** Mustafa Takesh, Khaldoun Odat Allh, Stefan Adams, Christian Zechmann

**Affiliations:** ^1^Department of Nuclear Medicine, Heidelberg University Hospital, Im Neuenheimer Feld 400, 69120 Heidelberg, Germany; ^2^Department of Urology, Heidelberg University Hospital, Im Neuenheimer Feld 110, 69120 Heidelberg, Germany; ^3^Department of Nuclear Medicine and Radiology, Knappschaft Hospital, 66280 Sulzbach, Germany

## Abstract

^18^F-FECH-PET/CT has been proved to be an imaging agent for prostate carcinoma. However, its role in detecting the bone metastases is still blurred owing to the lack of related studies. The purpose of our study was to assess the efficacy of PET with ^18^F-ethylcholine in assessing the bone status and to compare the results with that of conventional bone scan findings. For this purpose, we selected 37 patients (mean age 69 ± 7), who had been referred for restaging purposes due to biochemical recurrences and underwent both ^18^F-FECH-PET/CT and bone scan in a short interval. Generally 18 patients out 37 patients referred with biochemical relapse were confirmed to have bone involvement. From 18 confirmed bone involvement cases, ^18^F-FECH-PET/CT identified correctly the bone involvement in 15 cases with overall sensitivity of 83.3%. On the other hand, bone scan identified 17 out of 18 confirmed cases with overall sensitivity of 94.4%. The lesion-related results show that the sensitivity of each investigation differs with the anatomical regions, and by comparing both results, ^18^F-FECH-PET/CT was mostly superior to bone scan; however, without a statistical significance (*P* > 0.1). In conclusion, no significant gain in sensitivity was achieved using bone scan compared with ^18^F-FECH-PET/CT.

## 1. Introduction

Carcinoma of the prostate is the most common malignancy in men with increased incidence rates owing to the population ageing and the improvement of diagnostic procedures. 

The early detection of the bone metastases is of value in making decision regarding the treatment plan, which may vary extremely according to the bone status.

The likelihood of the bone metastases at the first diagnosis varies with the histological score and serum level of prostate-specific antigen, and it is possible to some extent to estimate the degree of tumor spread in the light of the PSA value. Bone involvement is much less frequently involved with low PSA level, so that the bone staging is not recommended with PSA less than 10 ng/mL, except if there are known bone disorders which may later cause false positive findings [1–3]. However, in patients with PSA relapse after primary therapy it is not easy to be guessed based on PSA values whether the bone involvement or another involvement is behind a PSA rising.

In established skeletal involvement, there is a need of effective imaging method to monitor the status progress. Bone scan is the widely used screening technique for assessing the bone status in the most malignancies, and it shows mostly high sensitivity in detecting the bone involvement. However, it faces a serious disadvantage represented by the low specificity. PET/CT with ^18^F-fluorodeoxyglucose (FDG) is known to have general limitation in prostate cancer. PET with radiolabeled choline such as ^18^F-FECH-PET was found to be an effective method in diagnosis of primary and recurrent PCA tumors as well [4, 5]. Two possible mechanisms have been proposed to explain the increased choline uptake in prostate cancer cells. The first is increased cell proliferation in tumors, because choline is a precursor for the biosynthesis of phosphatidylcholine and other phospholipids, the major components of the cell membrane. The second explanation is the overproduction of choline kinase in cancer cells, which was experimentally confirmed in human-derived prostate cancer [6–8]. 

Although, ^18^F-FECH-PET is being increasingly used primarily in patients with prostate cancer and for followup, its efficacy in detecting the bone involvement still needs further investigations in comparison with the established method (bone scan). To our knowledge there are little studies discussing this topic.

We aim in this study mainly to assess the value of ^18^F-FECH-PET/CT in detecting the bone metastases and to compare the results with that of bone scan.

## 2. Materials and Methods

### 2.1. Patients and Art of Therapy

37 patients (mean age 69 ± 7) had been referred in our department for re-staging purposes due to biochemical recurrence, PSA median 2.6 ng/dL (range 0.3–21). Gleason's score ranges among 3 and 9: GS.7 (*n* = 12), GS.8 (*n* = 8), GS.9 (*n* = 7), GS. 3 (*n* = 1), and GS 5 (*n* = 1). Gleason's score was unavailable in eight patients.

The patients underwent a wide variety of initial therapies including radical prostatectomy (*n* = 11), radiotherapy (*n* = 7), and pure hormonotherapy in patients with locally advanced disease (*n* = 3). A number of patients underwent combined therapy due to previous slight PSA increase such as radical prostatectomy followed by salvage radiotherapy (*n* = 12), radical prostatectomy followed by salvage antihormonal therapy (*n* = 3), or radical prostatectomy followed by HIFU (*n* = 1). Nine patients were under ongoing systemic therapy (chemotherapy or antihormonal therapy). 

The study was approved by the ethics committee of the Heidelberg University, and a written consent was obtained from all patients. 

### 2.2. Imaging Techniques

Bone scintigraphy was performed using a modern double-head Gamma-camera (Ecam, Siemens Medical Solution) equipped with low-energy, high-resolution collimators. Whole-body images were obtained 2-3 h after intravenous injection of 700 MBq (19 mCi) of ^99m^Tcm-MDP at the scan speed of 15 cm/min in the anterior and posterior projection; additional imaging were acquired, when required.

All PET studies were carried out using a Biograph 6 PET/CT (Siemens/CTI). Imaging was started as dynamic imaging immediately parallel to administration of a standard of 250 MBq of ^18^F-ethylcholine for 10 min before proceeding to whole body imaging. For attenuation correction of the PET scan, a low-dose CT (130 keV, 30 mAs; Care Dose) without contrast medium was done. Static emission scans, corrected for dead time, scatter, and decay were acquired from the vertex to the proximal legs requiring eight bed positions, 4 minutes each. The images were iteratively reconstructed with the OSEM algorithm using four iterations with eight subsets and Gauss filtering to an in-plane spatial resolution of 5 mm at full-width half-maximum. 

Both tests were done in a short interval. The delay between both modalities ranged between 2 and 45 days (average 14 days).

The images were evaluated visually by two skilled nuclear medicine physicians in presence of a skilled radiologist after viewing the images in different planes. The image assessment was achieved in combination with the clinical information and close correlation with other available imaging studies.

### 2.3. Correlation Methods

#### 2.3.1. Patient Based Results

The findings concerning the bone status were assessed as positive in presence of typical findings validated with followup and/or by correlation with other modality and as negative in absence of the pathological bone uptake (there was no need to prove the negativity), or false positive/negative (if proved the contrary). Sensitivity, specificity, NPV, and PPV are the statistical indicators utilized in assessing the universal outcome.

#### 2.3.2. Lesion-Based Results

**Figure 1:**
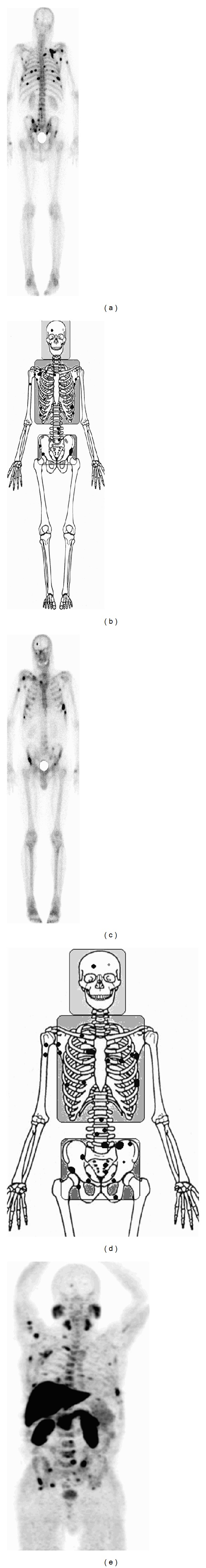
Comparison method between ^18^F-FECH-PET and BS (bone scan) (a). Bone scan (dorsal view). (b) Corresponding skeleton outline of bone scan. (c) Bone scan (ventral view). (d) Corresponding skeleton outline of ^18^F-FECH-PET. (e) ^18^F-FECH-PET (MIP).

In the patients with confirmed bone metastases (*n* = 18), the skeleton was divided into five regions (skull, ribs, pelvis, vertebra, and extremities) to simplify the comparison between the matching regions in both examinations. The metastases had been drawn as black points on a separate skeleton outline (Figure 1). Each imaging was evaluated separately, so that the interpretation would not be influenced. 

The ANONA test was used to compare the extent of metastatic spread in both examinations in the different regions.

### 2.4. Choline Uptake Correlated Quantitatively with Both MDP Uptake and Hounsfield's Units

SUVs were acquired by manually drawing a volume of interest over the pathologic bone lesions that had been identified on visual analysis of static emission images. By using attenuation-corrected PET data, SUVs were calculated as the ratio of regional radioactivity concentration (becquerel/milliliter) divided by the injected amount of radioactivity (Becquerel) normalized to body weight in gram. 

CT reading was based on characteristic patterns of morphological change; all detectable lesions on CT were categorized by a radiologist as sclerotic, lytic, or mixed metastases. The quantitative radiodensity of sclerotic lesions was measured by means of the Hounsfield unit (HU).

For quantitative assessment of MDP uptake, we considered an uptake score based on the visual assessment and graduated from 0 till 4 (0 no uptake, 1 decent uptake, 2 moderate uptake, 3 high uptake, 4 extremely severe uptake). 

So the intensity of choline uptake was once compared with the radio-density represented by HU and once with MDP uptake (evaluated visually).

Lesions detected in CT and evaluated as high suspected were encompassed in this comparison, regardless whether they showed a positive choline uptake or not.

Some limitations should be kept in mind such as the absence of pathological verification of the bone metastases. Thus, the patients were confirmed as having bone metastases just depending on clinical followups in correlation with other diagnostic tools, for example MRI. The average clinical followup duration to confirm the final diagnosis was 1 year. Moreover, the histopathological confirmation is a common problem shared with such clinical studies.

## 3. Results

The characteristics of the patients are summarized in Table 1.

### 3.1. Overall Results

Generally, 18 patients out 37 patients referred with biochemical relapse were confirmed to have bone involvement. 

**Figure 2:**
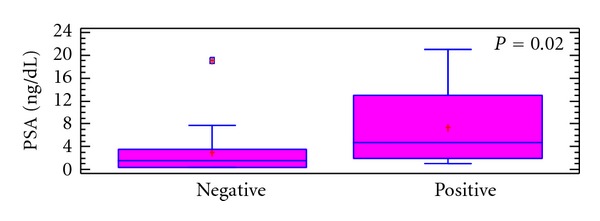
Comparison of PSA values in patients with negative bone findings and those with positive bone findings (paired *t*-test, *P* = 0.02). The statistic is summarized as a box plot.

Since the pathological confirmation was not available, the followup and correlation with other modalities were adopted in our verification. (Followup was available in 11 patients; in the remaining seven patients, the verification was based on a typical findings in PET, assessed by two nuclear medicine physicians matching with morphological images such as CT or MRI.) In patients with positive bone findings, the PSA median was 4 ng/dL (range 1–21). By contrast, the patients with negative bone findings had a PSA median value of 1. 5 ng/dL (range 0.3–19). Statistically there was a significant difference between both groups (*P* = 0.02) (Figure 2).

In patients with negative bone findings the PSA relapse was attributed to lymph node metastases (*n* = 5), local recurrence (*n* = 2), and lung metastases (*n* = 1). The underlying cause is still unknown in 11 patients.

From 18 confirmed bone involvement cases, ^18^F-FECH-PET/CT identified correctly the bone involvement in 15 cases with a sensitivity of 83.3%. No false positive was identified; consequently its specificity was 100%, NPP 86.3%, and PPV 100%.

On the other hand, bone scan showed the presence of the metastases in 17 out of 18 confirmed cases with sensitivity of 94.4%. The single false negative finding was due to a bone marrow metastasis.

From 19 negative findings, the bone scan showed two false positive cases due to Paget disease and trauma. Hence with a specificity of 89.4%, PPV of 89.4% and NPV of 94.4%.

### 3.2. The Area-Based Results

As mentioned earlier, the skeleton had been divided into multiple regions to simplify the matching of findings. 

Totally, the patients with positive findings have 122 lesions. ^18^F-FECH-PET was capable to identify 101 lesions with a sensitivity 82.7% versus 109 identified using BS (bone scan) with sensitivity of 89.4%. The sensitivity of both modalities differs extremely with the anatomical region (Table 2).

**Figure 3:**
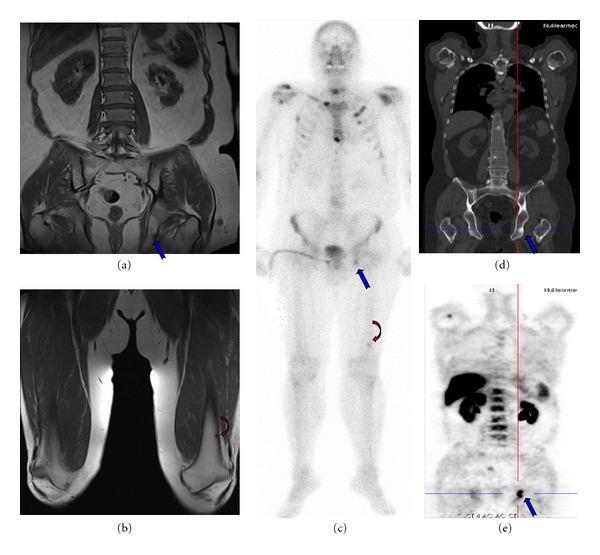
61-year-old prostate cancer patient with confirmed bone metastases; T1-weighted coronal plane (a) + (b); bone scan (c); ^18^F-FECHPET/CT Imaging (d) + (e). The images show a correlation between all modalities in demonstrating the metastasis in pelvis. However, the ^18^F-FECH-PET failed in showing the femur metastasis (outside the field of view—FOV).

Indeed this superiority of bone scan was demonstrated in ribs (71% in ^18^F-FECH-PET versus 94% in BS) and cranium. In remaining regions, ^18^F-FECH-PET was superior in identifying more metastases, in vertebrae (92% versus 85%) and pelvis (91% versus 89%). In the extremities ^18^F-FECH-PET shows also superiority over bone scan (90% versus 50%) in spite of limited field of view (FOV) Figure 3.

Statistically using ANOVA test there was no significant difference between both modalities (*P* > 0.1) in the number of detected metastases in all anatomical regions.

#### 3.2.1. Examples of Superiority of ^18^F-FECH-PET/CT Compared with BS in Specificity in Traumatic and Degenerative Alterations

**Figure 4:**
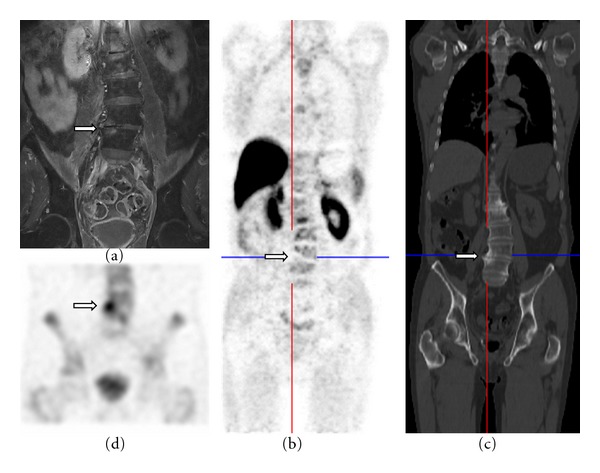
(a) MRI T2-weighted coronal plane shows a degenerative change in the lumbar spine at the level of L4/5. (b), (c) ^18^F-FECH-PET/CT (coronal planes). (d) Bone scan (SPECT).

**Degenerative Changes: ^18^F-FECH-PET/CT versus BS (Figure 4)**

Whereas the bone scan demonstrates an intensive uptake in this degenerative change, the ^18^F-FECH-PET/CT shows a negative choline uptake.

**Figure 5:**
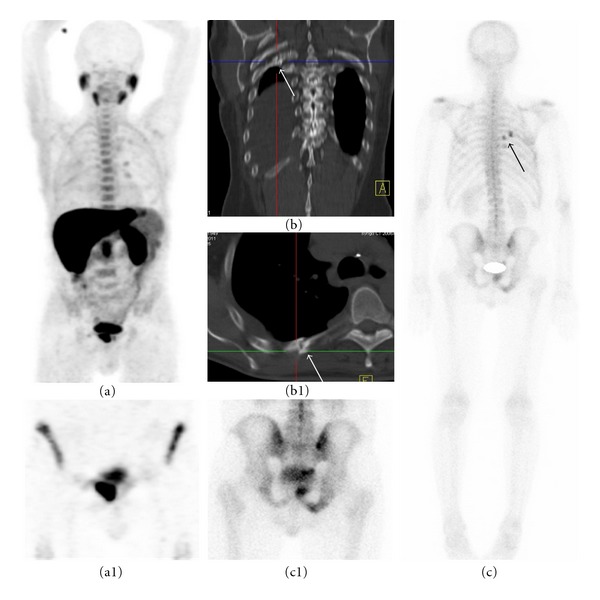
62-year old patient referred with PSA elevation and underwent both bone scan and ^18^F-FECHPET with interval of four days. ((a), (a1)) ^18^F-FECHPET (MIP) and coronal plane in the pelvis show a bone involvement in the pubis. ((b), (b1)) coronal and axial planes of CT thorax shows rib fracture. ((c), (c1)) bone scan (posterior view) and spot image of pelvis show bone metastases in the right pubis in addition to two hot spots in 6th and 7th rib in the right hemithorax corresponding with ribs fracture. By contrast, ^18^F-FECH-PET shows negative uptake in these fractures.

**Figure 6:**
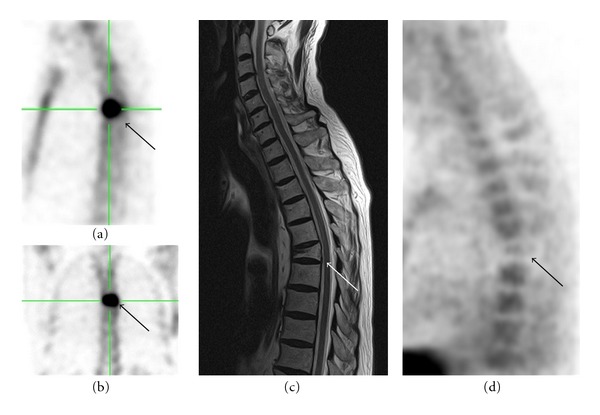
((a), (b)) Bone scan, sagittal and coronal planes, respectively, show an intensive uptake in middle thoracic spine. (c) MRI-T1 weighted shows a compression fracture at the same location. (d) ^18^F-FECH-PET shows relatively an uptake reduction.

**Trauma: ^18^F-FECH-PET/CT versus BS (Figures 5 and 6)**

These images show a discrepancy between MDP uptake and choline uptake in a compression fracture and rib fractures.

A total of 94 out of 122 with the following distribution: (3 in cervical spine, 10 in lumbar spine, 35 in pelvis, 7 in ribs, 4 in sternum, 15 in thoracic spine, 12 in the extremities and 7 in other regions) were selected to make a further quantitative comparison between ^18^F-FECH-PET and both CT and bone scan using the above mentioned quantitative parameters. 28 lesions were excluded from this analysis, for example, in ribs due to neighborhood to liver (false high SUV through partial volume effect) or due to technical limitations. As previously mentioned, the quantitative assessment in bone scan was based on visual score escalating from 0 till 4.

Based on the radiological portrayal in CT, lesions were divided into osteoblastic lesions (*n* = 42), osteolytic (*n* = 19), and mixed (*n* = 16).

**Figure 7:**
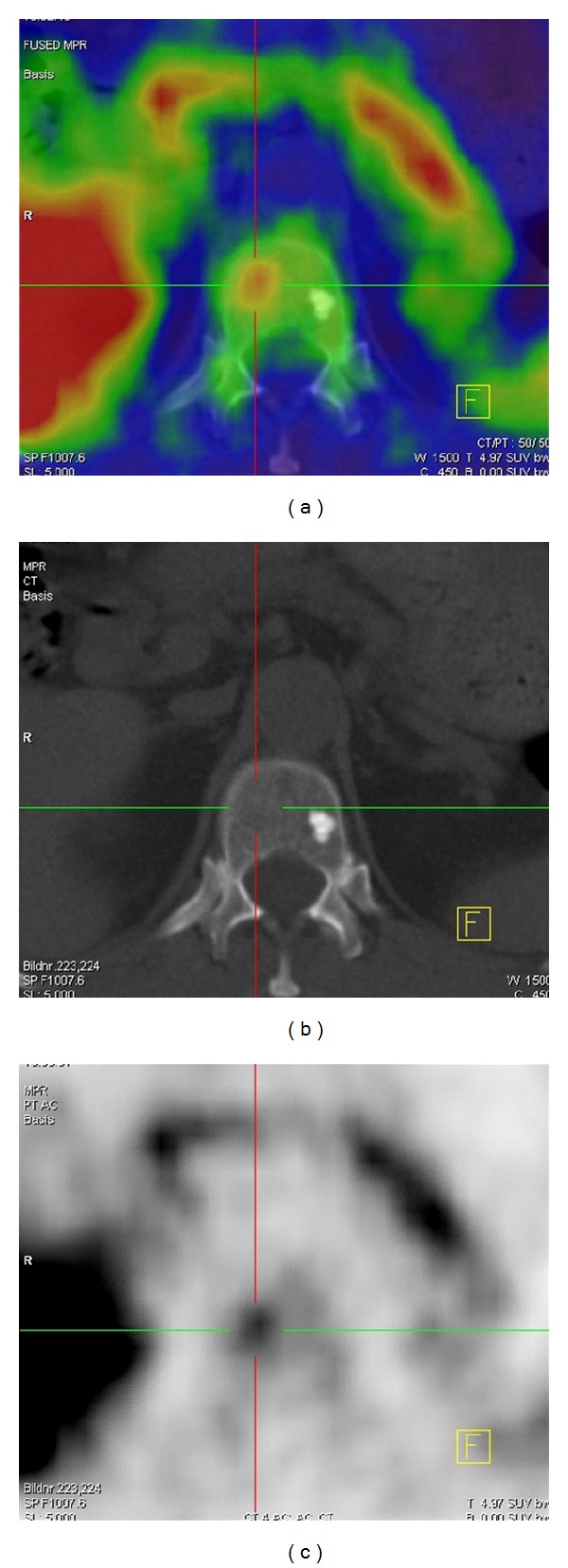
Axial planes of ^18^F-FECH-PET/CT in lower thoracic spine show intensive choline uptake ((a), (c)), attributed to bone marrow involvement without morphological alteration (b). In contrast, in the neighborhood there is an osteoblastic metastasis showing a negative choline uptake.

16 lesions of positive choline uptake: (11 in the spine—7 thoracic and 4 lumbar—2 in the pelvis and 3 in the extremities) were lacking morphological alterations. They were characterized with high choline uptake (SUV 5, 95 ± 1, 5) and mostly with decent or absent MDP uptake. They were attributed to bone marrow involvement (Figure 7) 13 of sclerotic lesions (6 in pelvis, 3 in thoracic spine and 4 in ribs) (HU mean 739 ± 216) were of negative choline uptake but mostly of intensive MDP uptake (qMDP score ≥3) (qMDP quantitative MDP evaluated visually).

**Figure 8:**
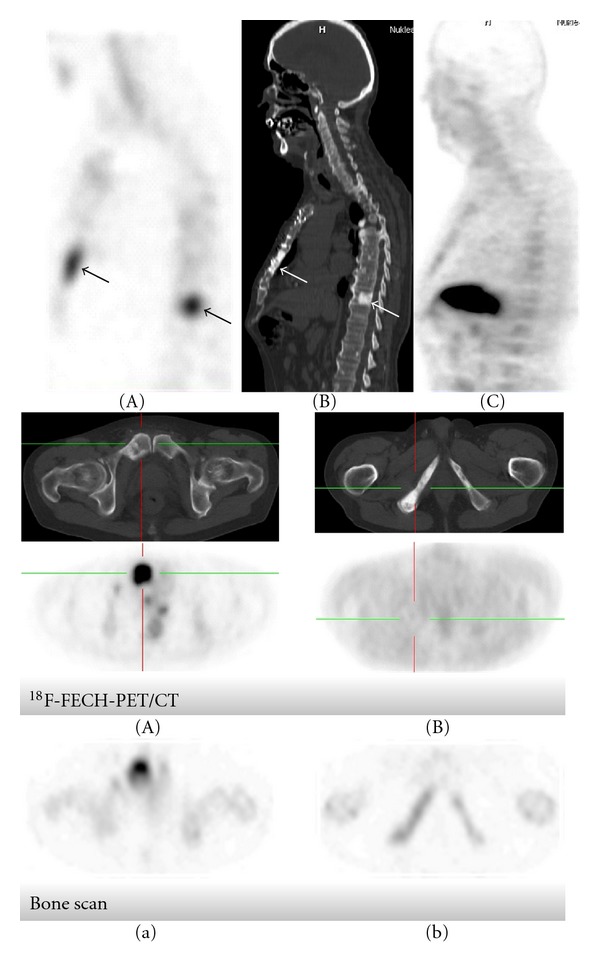
62-year old patient with confirmed PCA referred with PSA elevation in spite of ongoing AHT; he underwent both BS and ^18^F-FECH-PET/CT within 4 days. In the upper image, (A) bone scan sagittal plane, (B) CT thorax sagittal plane, (C) ^18^F-FECH-PET/CT sagittal plane. We see clearly a negative choline uptake in the osteoblastic metastases in sternum and thoracic spine, whereas bone scan demonstrates them. In the lower images, ^18^F-FECH-PET/CT shows a metastasis in the pubic body matching with an osteolytic bone lesion (A); at this site the bone scan shows a severe MDP uptake (a). Additionally, the bone scan shows a further positive finding in the inferior ramus of pubis (b) corresponding with sclerotic metastasis however without choline uptake (B). This image shows the osteolytic metastasis was kept out of AHT impact.

The decline or absence of choline uptake in sclerotic metastasis was noted in particular in patients receiving systemic therapy (Figures 8 and 11). 

In contrast, the osteolytic metastases (4 in the pelvis and 1 in the humerus) were characterized with high choline uptake (7 ± 2, nadir 5.5) and wide extent of MDP uptake (4 showed negative uptake, 4 showed intensive uptake and 2 showed moderate uptake).

**Figure 9:**
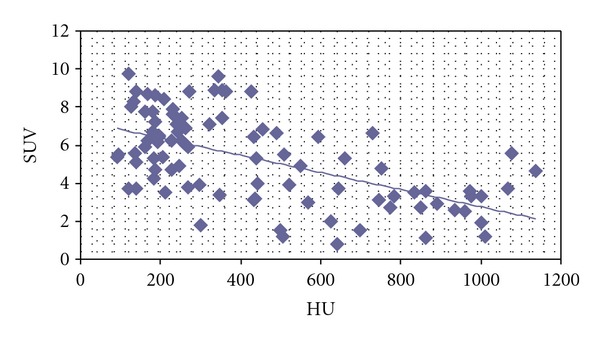
The relationship between choline uptake measured by SUV (max) and the density of sclerotic measured by the Hounsfield units (HU) in bone metastases, which was presented in a scatter plot showing a moderate negative correlation with statistical significance (*r* = −0.58, *P* < 0.01).

A significant negative correlation was demonstrated between tracer uptake SUV(max) and the density of sclerotic lesions assessed by HU (*r* = −0.58, *P* < 0.01) (Figure 9).

On the other hand, numerical score of MDP was correlated negatively with SUV(max) value (*r*  −0.01) and positively with HU (*r* 0.34). However, they were statistically not significant. 

**Figure 10:**
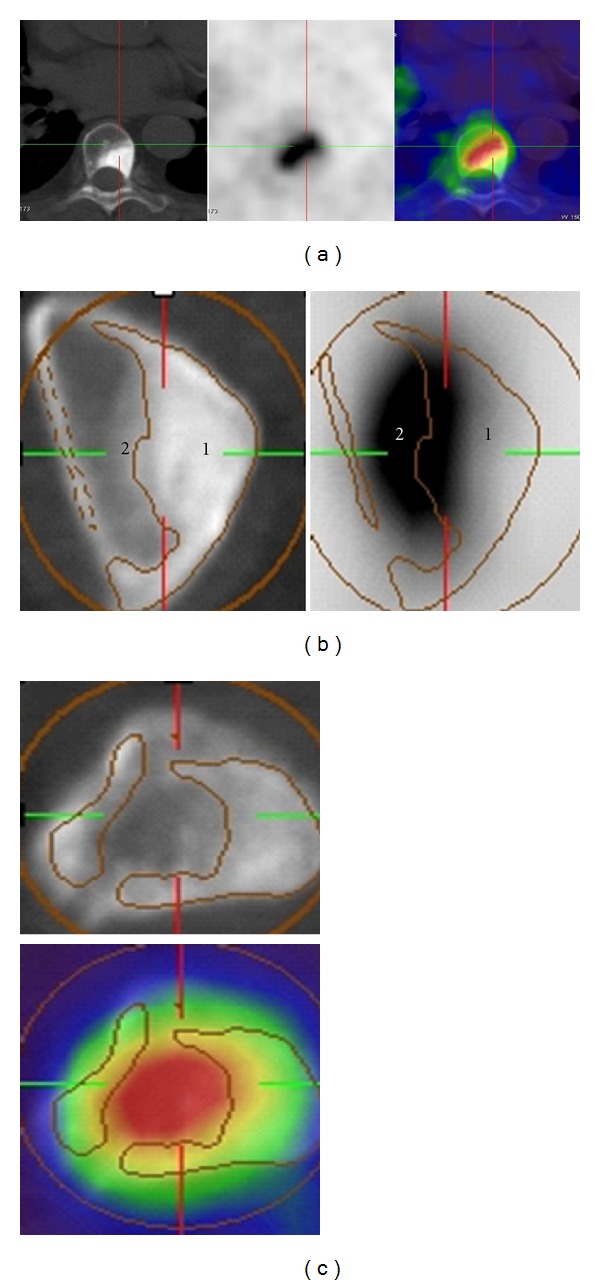
Shape of choline uptake in sclerotic and mixed bone metastases. (a) Osteoblastic metastasis in vertebra: the radioactivity concentrates in the rim (low sclerosis level). (b) Osteoblastic metastasis in ischium: we notice negative uptake in the area of metastasis with a high sclerosis (1); here the activity from part 2 can spill into part 1, which in turn will lead the examiner to think that there may be more viable tumor tissue within the osteoblastic part than there really is. (c) Mixed metastasis: we notice the radioactivity concentration in osteolytic part.

 In few mixed metastases it was demonstrated that the choline uptake was solely concentrated in the osteolytic part evading the sclerotic part (Figure 10(c)). In pure sclerotic metastases it was shown that uptake concentrates at the rim of metastases, where there is a transitional region with a moderate sclerosis (Figures 10(a) and 10(b)).

**Examples of the Effects of Ongoing Systemic Therapy on PET Findings**

**Figure 11:**
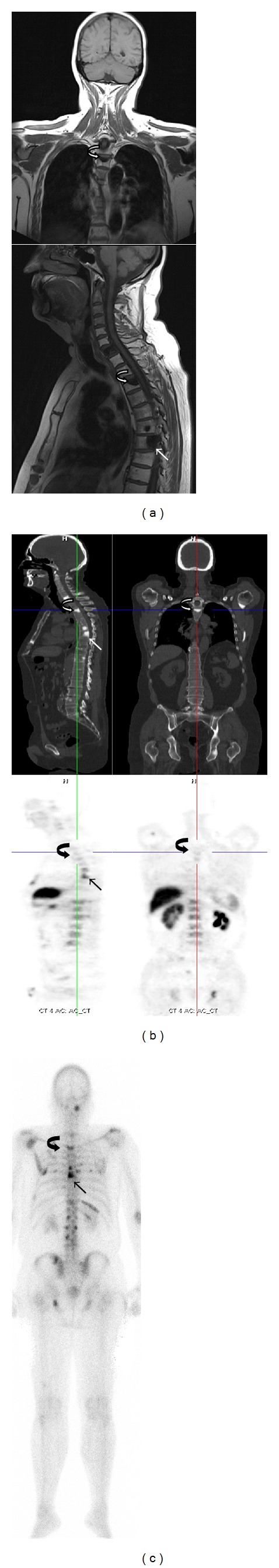
Inhomogeneous therapy effect on the different bone metastases: 63-year-old PCA patient with known bone metastases was referred shortly after chemotherapy onset. (a) MRI T1-weighted coronal and sagittal planes. (b) ^18^F-FECH-PET/CT, coronal and sagittal planes (c). Bone scan (posterior view). In the upper thoracic spine (curved arrow) both CT and MRI show bone metastasis without PET correlation ((b) lower images). By contrast, bone scan demonstrates this metastasis clearly. In middle thoracic vertebrae (straight arrow), both CT and MRI show another bone metastasis; it is of positive choline and MDP uptake.

**Figure 12:**
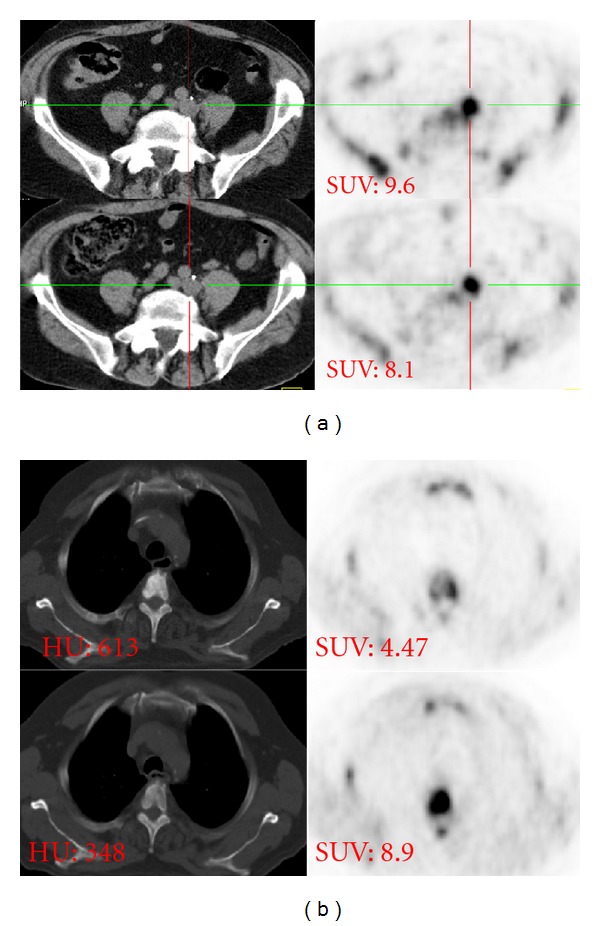
73-year old patient with prostate cancer metastatic to regional lymph nodes (a) and thoracic spine (b). Lower row: baseline Upper row: followup after 6 months under AHT. Bone metastasis (b) shows a dramatic SUV (max) decreasing (8.9 versus 4.47); however, the associated CT shows an increasing of sclerosis degree (HU 348 versus 613) suggesting progressive disease in spite of SUV decline. The lymph node metastasis (a) shows a SUV(max) rising (8.1 versus 9.6), confirming thus the progress.

See Figures 11 and 12.

## 4. Discussion

It is known that the skeleton is a favorable place for metastasis in prostate cancer. It is the second most common site of metastatic disease after lymph nodes and considered as the main cause of morbidity and mortality in prostate cancer patients and mostly related to poor prognosis. The early detection of bone involvement is very crucial for appropriate management. Although bone scan is ranked first in assessing bone status in many malignancies including PCA, it still lacks specificity. 

The comparison between PET modality and bone scan in detecting bone involvement was a concern of many studies, mostly involving ^18^F-FDG PET/CT [9–11]. Cheng et al. [12] showed, in their review of six studies, that ^18^F-FDG PET/CT has both higher sensitivity and specificity than bone scintigraphy.

In patients with PCA, Tiwari et al. [13] proved that ^18^F-FDG-PET/CT can play a complimentary role to the conventional skeletal scintigraphy, particularly in detection of bone marrow disease. 

Whether or not ^18^F-FECH-PET/CT is capable to assess the bone status in an adequate way or requires supplementary test is still blurred. According to our knowledge, there is lack of studies concerning the comparison of ^18^F-FECH-PET/CT with standard BS. Beheshti et al. [14] showed that ^18^F-choline- PET/CT had lower sensitivity than ^18^F fluoride PET-CT for detection of bone metastases (^18^F-fluoride is bone seeking PET radiopharmaceutical).

Bone scan showed superiority over ^18^F-FECH-PET/CT in patient-based results (sensitivity 81.2% versus 93.7%). However, the high sensitivity of bone scan encounters a high likelihood of false positive findings. By contrast, ^18^F-FECH-PET/CT appears to be less affected by such unspecific lesions. The benefit of associated diagnostic CT (occasionally with contrast media) should be kept in mind in minimizing the false positive cases. Yet, CT is not the sole factor affecting the increase of the specificity of ^18^F-FECH, McCarthy et al. [15] suggested that ^18^F-FECH-PET can separate benign conditions such as trauma and arthropathy from malignancy and may be useful as an assistant to bone scan in equivocal cases. 

Basically, there is need to perform bone scan in case of negative ^18^F-FECH-PET/CT finding if there is clinical suspicion, under suggestion that ^18^F-FECH-PET is of less sensitivity. However, bone scan can be reserved and an unnecessary radiation exposure can be avoided, if proven otherwise. So whether or not bone scan can be abandoned as a complementary test to ^18^F-FECH-PET/CT is of a high clinical value.

We found that ^18^F-FECH-PET/CT showed less sensitivity compared with BS; however, two out of three patients demonstrating false negative PET findings were under systemic therapy, suggesting a potential impact of systemic therapy in minimizing the sensitivity. 

### 4.1. Sensitivity Variance in Different Regions

In our patient group, ribs and pelvis were the most commonly involved areas in about 60% of patients.

Looking at the findings of both modalities in the various anatomical regions, we observed better lesion detection efficiency of BS in the ribs with sensitivity of 98% versus 73%. On the other hand, the ^18^F-FECH-PET/CT was more effective in pelvis (93% versus 87%) and in extremities in spite of the presence of few metastases outside the field of view. In vertebra the ^18^F-FECH-PET/CT was also more accurate than BS. 

Because of poor spatial resolution of BS, an uptake in the spine could be incorrectly attributed to degenerative changes that may explain the low sensitivity of BS in spine compared with ^18^F-FECH-PET/CT. Moreover, a single lesion may be overlooked in planar BS. Indeed, SPECT (single photon emission computed tomography) can minimize the shortcoming of planar BS in the assessment of the spine; however it is not routinely applied.

In this regard, it should be mentioned that spinal metastases have typical locations, mostly in the posterior part of vertebrae due to the many short intraosseous arteries [16, 17], and they can be easily recognized and differentiated from degenerative changes in ^18^F-FECH-PET/CT in the light of morphological view provided by CT component.

In other anatomical regions ^18^F-FECH-PET/CT showed advantage over BS; this can be attributed to the superior spatial resolution. Of course the associated CT contributed to a certain degree in increasing the sensitivity of ^18^F-FECH-PET/CT through detecting the bone metastases of a negative choline uptake. These metastases were mostly of sclerotic type. In the same topic, we found that FECH uptake decreases with increasing sclerosis (negative correlation between HU and SUV(max) *r* = −0.58, *P* < 0.01). Moreover antihormonal therapy (AHT) was of selected impact in sclerotic metastases. This issue emphasizes the importance of CT in compensating the deficiency of ^18^F-FECH-PET in patients under AHT.

Further benefit of CT is the validating the PET finding through excluding other benign changes that may share the same findings with the metastases.

### 4.2. Bone Marrow (BM) Involvement in Both Modalities

Since the bone marrow is associated with minimal morphological changes and unusual to cause bone reaction, it remains undetectable or can be easily overlooked using morphological modalities or using bone seeking nuclides. Kato and coworkers reported a case of bone marrow metastasis finally detected by marrow biopsy, without any abnormality on bone scintigraphy or computed tomography [18].

Generally, the BM aspiration and biopsy remain the procedures of choice to detect BM involvement. ^18^F-FDG PET is supposed to have special value in detecting BM involvement; Aydin et al. [19] described a case of bone marrow metastasis detected by ^18^F-FDG PET and missed by bone scintigraphy in widespread melanoma. ^18^F-FECH-PET/CT in prostate cancer patients shares this property, whereas there is no role of CT or BS. Beheshti et al. [20] described a positive choline bone marrow metastasis in the thoracic spine without any CT abnormality.

In our results, PET positive findings without morphological changes in spine, pelvis and proximal extremities were attributed to bone marrow involvement. BS was not of further benefit in identifying them; they were either of negative or decent MDP uptakes. That raises the following question: What is the real clinical benefit of BS over ^18^F-FECH-PET/CT, if the deficiency of choline-PET is mainly in osteoblastic metastases, which are simply to be visualized using associated CT? In other words, if CT can adequately compensate the deficiency of ^18^F-FECH-PET, still bone scan offers further diagnostic information? Indeed, in order to validate its further performance as complementary test, bone scan is required to show a superiority over ^18^F-FECH-PET/CT both components PET and CT. Just looking at lesion-based results, there is no real justification for additional bone scan, since the gain in detecting more lesions is not valuable in patients with multiple metastases, who should anyway undergo systemic therapy. However, in patient-based results we notice that associated CT failed in identifying three false negative cases in ^18^F-FECH-PET/CT, which were ultimately detected using BS. More advantage of bone scan is emphasized in its availability and ease to repeat for followup. Moreover, in multiple bone metastases if pain radiotherapy is indicated, bone scan is necessary because MDP distributes in a similar way as bone seeking therapy-tracer.

### 4.3. Impact of Systemic Therapy

In ^18^F-FECH-PET/CT, choline uptake is supposed to be linked with presence of tumor cells which can transport and metabolize it. In contrast, BS displays the bone metastases indirectly through demonstrating the structural alterations. For this reason, any alteration concerning the tumor focus (whether progress or regress) is supposed to be first detected in choline-PET. This fact and the possibility to perform a quantitative assessing grant the ^18^F-FECH-PET a unique value in therapy monitoring. 

Subsequent to therapy onset, a drop in SUV is an appropriate indicator for response. By contrast, in bone scan the therapy response may lead to transient increasing MDP uptake due to an amplified osteoblastic response in the so-called “flare phenomenon.” This can be mistaken with progress and typically lasts about 6 months after therapy [21–23]. For this reason, the early response can never be assessed using BS. CT is also of negligible role in therapy monitoring, since the morphological alterations occur in delay. 

To understand the weakness of BS in therapy monitoring, we ought to return to the pathology of the bone metastases in prostate cancer. In this type of bone metastasis, intramembranous ossification takes place in regions of fibrous stroma and after therapy administration; the ossification process stays for a certain time running in the manufactured stroma despite damaging the causing tumor cells and evoked mediators. In other words, the ongoing mineralization processes, thus MDP uptake, would not be inhibited after therapy onset even if the therapy is successful.

Compared with BS, as mentioned earlier, choline uptake in ^18^F-FECH-PET/CT is supposed to reflect directly the tumor focus, and the reduction in SUV early after initiation of therapy can distinguish responders from nonresponders. However, does uptake decline always indicate response? In some cases, it was demonstrated that shortly after treatment onset, the ^18^F-FECH-PET/CT turned to be negative just in osteoblastic metastases, while osteolytic metastases were preserved. This effect of AHT in the sclerotic metastases was already described by Beheshti et al. [20]. It may also explain the lower total sensitivity of ^18^F-FECH-PET/CT compared with BS in the area-based results (6 patients were under ongoing AHT in our study).

In another case of our study under AHT, the followup shows a dramatic uptake decline in bone metastases. However, there is uptake increase in the accompanying lymph node metastases. Therefore this uptake decline in bone metastases is likely to reflect a temporal diminishing of uptake rather to be an indicator for real response. A similar case was described by Beheshti et al. [20].

The loss of choline uptake was also observed in osteoblastic metastases in patients undergoing no therapy. As mentioned earlier, choline uptake declines with increasing sclerosis, probably due to the drop in blood supply thus in nuclide availability. Beheshti et al. [20] found that a HU level above 825 is associated with an absence of metabolic activity.

Due to the lack of long-term followup, it is still unknown whether or not these metastases are still alive if reaching the stage of negative choline uptake. Obviously, even if the metastasis center reaches this stage, the rim where there is a moderate sclerosis remains active. 

Ultimately, the potential role of ^18^F-FECH-PET/CT in therapy monitoring seems to be limited in case of bone involvement, because choline uptake is likely to be affected by local factors such as the level of sclerosis. 

## 5. Conclusion

No significant gain in sensitivity was achieved using bone scan compared with ^18^F-FECH-PET/CT. In lesion-based results, the diagnostic potential of both modalities varies in the different anatomical regions and shows ^18^F-FECH-PET/CT mostly of superior value. That was attributed to the higher spatial resolution and the additional benefit of accompanied CT except for its value in detecting the bone marrow involvement.

## Figures and Tables

**Table 1:** 

No.	Age	GS	Prior T	PSA	Ongoing T	FD	FECH-PET	BS	Interval
1	79	n/a	RP	3.01	NIL	LN-M	LN-M	Neg	4
2	64	7	RP + HIFU	6.5	NIL	LN-M	LN-M	Neg	14
3	75	n/a	RP + AHT	7.7	NIL	LN-M	LN-M	Neg	24
4	74	5	RTx	19	AHT	LN-M	LN-M	FP	0
5	60	3	RP	0.4	NIL	LN-M	LN-M	Neg	35
6	63	8	AHT	4.8	NIL	LR	LR	Neg	2
7	69	9	RP + AHT	1.9	NIL	LR	LR	Neg	11
8	76	n/a	RP	0.8	NIL	PM	PM	Neg	18
9	70	7	RP + RTx	3.6	NIL	NDR	Neg	Neg	38
10	59	9	RP + RTx	0.3	NIL	NDR	Neg	Neg	14
11	85	7	RTx	1.56	NIL	NDR	Neg	Neg	9
12	65	8	RP	0.66	NIL	NDR	Neg	Neg	3
13	73	n/a	RP	0.4	NIL	NDR	Neg	Neg	1
14	79	n/a	RP	0.4	NIL	NDR	Neg	Neg	1
15	68	n/a	RP + RTx	0.5	NIL	NDR	Neg	FP	25
16	68	7	RP + RTx	1.6	NIL	NDR	Neg	Neg	4
17	69	7	RP + RTx	1.9	NIL	NDR	Neg	Neg	3
18	68	9	RP + RTx	0.9	NIL	NDR	Neg	Neg	42
19	80	n/a	RTx	0.3	NIL	NDR	Neg	Neg	25
20	63	9	AHT	13.1	AHT	OSS	OSS	OSS	22
21	70	7	RP	1	NIL	OSS	OSS	OSS	45
22	71	7	RP + RTx	12.9	NIL	OSS	OSS	OSS	5
23	77	8	RP + RTx	1.8	RTx	OSS	OSS	OSS	24
24	69	8	RP + RTx	1.7	NIL	OSS	OSS	OSS	9
25	56	9	RP + RTx + AHT	6.7	Docetaxel	OSS	OSS	OSS	4
26	71	8	RTx	2.9	AHT	OSS	OSS	OSS	15
27	59	8	RTx	3.63	NIL	OSS	OSS	OSS	11
28	61	8	RP	1.9	AHT	OSS	OSS	OSS	6
29	73	9	RP	21	NIL	OSS + LN-M	OSS + LN-M	OSS	7
30	71	8	RTx	5	AHT	OSS + PM	OSS + PM	OSS	45
31	66	7	RP + RTx	21	Docetaxel	OSS + LN-M	LN-M FN	OSS	14
32	64	7	RP + AHT	1	AHT	OSS + LN-M	LN-M FN	OSS	7
33	76	7	RP	5.6	NIL	OSS	FN	OSS	3
34	67	7	RTx	4.5	NIL	OSS	OSS	FN	18
35	65	7	RP	12.1	NIL	OSS	OSS	OSS	2
36	62	9	AHT	14	AHT	OSS	OSS	OSS	11
37	77	n/a	RP + RTx	4	NIL	OSS	OSS	OSS	5

PR: radical prostatectomy, RTx: radiation therapy, CTx: chemotherapy, NIL: nothing, AHT: hormone/androgen deprivation therapy, LN-M: lymph node metastasis, OSS: bone metastasis, PM: pulmonary metastasis, Neg: negative finding.

**Table 2:** The sensitivity of both modalities in different skeleton regions.

Anatomical regions	Bone scan	FECH-PET/CT	Total number	Sensitivity (bone scan)	Sensitivity (FECH-PET/CT)
Cranium	5	4	5	100%	80%
Spine	12	13	14	85%	92%
Ribs	54	40	56	96%	71%
Pelvis	33	34	37	89%	91%
Extremities	5	10	10	50%	100%

Totally	109	101	122	89.3%	82.7%

## Abbreviations

^18^F-FECH-PET: F-fluorethylcholine-PET
PCA: Prostate cancer
BS: Bone scan
MRI: Magnetic resonance imaging
PPV: Positive predictive value
NPV: Negative predictive value
PR: Radical prostatectomy
RTx: Radiation therapy
CTx: Chemotherapy
NIL: Nothing
ANT: Hormone/androgen deprivation therapy
LN-M: Lymph node metastasis
OSS: Bone metastasis
PM: pulmonary metastasis
Neg: Negative finding.
